# Intrapericardial Delivery of Cardiosphere-Derived Cells: An Immunological Study in a Clinically Relevant Large Animal Model

**DOI:** 10.1371/journal.pone.0149001

**Published:** 2016-02-11

**Authors:** Rebeca Blázquez, Francisco Miguel Sánchez-Margallo, Verónica Crisóstomo, Claudia Báez, Juan Maestre, Verónica Álvarez, Javier G. Casado

**Affiliations:** 1 Stem Cell Therapy Unit, ‘Jesús Usón’ Minimally Invasive Surgery Centre, Cáceres, Spain; 2 Endoluminal Therapy and Diagnosis, ‘Jesús Usón’ Minimally Invasive Surgery Centre, Cáceres, Spain; Centro Cardiologico Monzino, ITALY

## Abstract

**Introduction:**

The intrapericardial delivery has been defined as an efficient method for pharmacological agent delivery. Here we hypothesize that intrapericardial administration of cardiosphere-derived cells (CDCs) may have an immunomodulatory effect providing an optimal microenvironment for promoting cardiac repair. To our knowledge, this is the first report studying the effects of CDCs for myocardial repair using the intrapericardial delivery route.

**Material and Methods:**

CDCs lines were isolated, expanded and characterized by flow cytometry and PCR. Their differentiation ability was determined using specific culture media and differential staining. 300,000 CDCs/kg were injected into the pericardial space of a swine myocardial infarcted model. Magnetic resonance imaging, biochemical analysis of pericardial fluid and plasma, cytokine measurements and flow cytometry analysis were performed.

**Results:**

Our results showed that, phenotype and differentiation behavior of porcine CDCs were equivalent to previously described CDCs. Moreover, the intrapericardial administration of CDCs fulfilled the safety aspects as non-adverse effects were reported. Finally, the phenotypes of resident lymphocytes and TH1 cytokines in the pericardial fluid were significantly altered after CDCs administration.

**Conclusions:**

The pericardial fluid could be considered as a safe and optimal vehicle for CDCs administration. The observed changes in the studied immunological parameters could exert a modulation in the inflammatory environment of infarcted hearts, indirectly benefiting the endogenous cardiac repair.

## Introduction

Clinical trials are continuously demonstrating that mesenchymal stem cells and resident cardiac stem cells are a promising cell source for regenerative therapy [1–5]. These cells fulfill the safety requirements being particularly attractive for their low immunogenicity, multipotentiality and self-renewal ability [1,6,7]. The route of administration, dose, time or cell type determine the success or failure of stem cell-based therapies and their therapeutic effect [8].

At the present, most of the preclinical studies have clearly demonstrated that the retention of transplanted cells in the heart is very low by any delivery method [9] and alternative techniques and administration routes need to be investigated to ensure the viability and differentiation potential as well as their homing and immunomodulatory capacity. Moreover, it would be desirable to guarantee the implantation of cells for a period of time enough to reach the desired therapeutic effect. In this sense, a higher retention rate may have a greater impact on cardiac repair enabling paracrine stimulation through the release of growth factors, pro-angiogenic molecules, immunomodulatory factors, proliferative and anti-apoptotic molecules.

Only a few reports address the question whether the intrapericardial delivery of adult stem cells could be a safe and effective alternative to other surgical procedures. The pericardial fluid (PF) composition is very similar to plasma and recent studies have demonstrated that it could be considered an optimal vehicle to preserve the viability, phenotype and proliferation of bone marrow-derived MSCs [10]. Moreover, in comparison to other routes, one positive aspect of pericardial delivery is that pericardial fluid has a low turnover rate that may provide a long term effect to achieve the desired therapeutic effect of stem cells.

Here we hypothesize that intrapericardial administration of cardiosphere-derived cells (CDCs) may have an immunomodulatory effect providing an optimal microenvironment for promoting cardiac repair. These CDCs have recently emerged as an effective cell type for cardiovascular cell therapy. Since the first report of cardiospheres in 2004 [11] and cardiosphere-derived cells in 2007 [12], several studies using clinically relevant large animal models have demonstrated the beneficial effect of these cells for the damaged cardiac tissue restoration. In these studies, the main administration routes assayed were the intracoronary infusion [13–15] and the intramyocardial injection [16]. Nowadays, clinical trials using CDCs are being conducted to test the efficacy of intracoronary-delivered CDCs [17–20]. To our knowledge, this is the first report studying the immmunomodulatory effect of intrapericardially delivered CDCs. More importantly, animals were followed up using magnetic resonance imaging, which is the gold standard for functional cardiac evaluation.

## Materials and Methods

### Isolation of porcine cardiosphere-derived cells

All experimental protocols were approved by the Committee on the Ethics of Animal Experiments of Minimally Invasive Surgery Centre and fully complied with recommendations outlined by the local government (Junta de Extremadura) and by the Directive 2010/63/EU of the European Parliament on the protection of animals used for scientific purposes. All surgery was performed under sevoflurane anesthesia, and all efforts were made to minimize suffering. Cardiosphere-derived cells (CDCs) were obtained from cardiac tissue explants of euthanized Large White pigs. Auricular explants (1–2 g) were washed with PBS and mechanically disrupted into 1–2 mm^3^ fragments. These fragments were washed again to eliminate cellular debris. The tissue was then subjected to three successive enzymatic digestions with a solution of 0.2% trypsin (Lonza) and 0.2% collagenase IV (Sigma) in PBS at 37°C for 5 min each. Digested tissue was washed with Complete Explant Medium (CEM) composed by 10% fetal bovine serum (FBS) (Sigma), 1% penicillin-streptomycin (Lonza), 2 mM L-glutamine (Lonza) and 0.2 mM 2-mercaptoethanol (Sigma) in IMDM (HyClone). Finally, explants were cultured in 90 mm Petri plates with CEM at 37°C and 5% CO_2_.

After three weeks, tissue fragments were discarded and fibroblasts-like cells migrating from tissue explants were trypsinized and seeded into 30 mm poly-D-lysine coated plates with Cardiosphere Growing Medium (CGM), composed by 10% FBS, 1% penicillin-streptomycin, 2 mM glutamine and 0.1 mM 2-mercaptoethanol in 35% IMDM and 65% DMEM-Ham’s F12 (Sigma). Under these conditions, suspended cells clusters called cardiospheres are formed, and cells migrating from them are the CDCs. These cells were selected, seeded again into culture flasks with CGM and expanded at 37°C and 5% CO_2_. CDCs at passages 5 to 10 were used for intrapericardial delivery.

### Phenotypic analysis of cardiosphere-derived cells by flow cytometry

For flow cytometric analysis, porcine CDCs were detached from culture flasks with 0.25% trypsin solution and suspended in PBS containing 2% FBS. The cells were then stained with FITC-conjugated monoclonal antibodies against human CD90 (porcine crossreactive) and FITC-conjugated porcine monoclonal antibodies against CD29, CD31, CD44, CD45, CD61, CD105, CD117, Sca-1, SLA-I (Swine Leukocyte Antigen class I) and SLA-II (Swine Leukocyte Antigen class II) from Serotec. The phenotypic analysis was performed as follows: 2×10^5^ cells were incubated for 30 min at 4°C with appropriate concentrations of monoclonal antibodies. The cells were washed and resuspended in PBS. The flow cytometric analysis was performed on a FACScalibur cytometer (BD Biosciences) after acquisition of 10^5^ events. Cells were primarily selected using forward and side scatter characteristics and fluorescence was analyzed using CellQuest software (BD Biosciences). Isotype-matched negative control antibodies were used in all the experiments. The mean relative fluorescence intensity was calculated by dividing the mean fluorescent intensity (MFI) by the MFI of its negative control.

### Molecular characterization of cardiosphere-derived cells by RT-PCR

To analyze the expression of different markers, total RNA from CDCs was isolated. For that, 1 mL of TRI-Reagent (Sigma) was added to the 24 well plates. Cells were transferred to an Eppendorf tube, 200 μL of chloroform were added and samples were incubated for 5–10 min at room temperature. After a centrifugation of 15 min at 12000 x g, the aqueous phase was mixed with 500 μL of isopropanol and incubated at -80°C for 20 min to precipitate the RNA. Consecutive centrifugations and ethanol washing were made. Finally, the pellet was resuspended in DEPC-treated water.

The cDNA was synthesized from 1 μg of RNA in reverse transcription reaction for 1 h at 37°C using Superscript III reverse transcriptase (Invitrogen). The sequences of the PCR primers (Table 1) were designed for *Sus scrofa* by using the NCBI Primer-BLAST tool (www.ncbi.nlm.nih.gov/tools/primerblast/).

**Table 1:** Sequences, melting temperatures, amplicon sizes and NCBI access numbers for the primers used in the PCR.

Gene	Primers sequences	T_m_ (°C)	Amplicon (bps)	Access number (NCBI)
**Stemness-related genes**				
KIT (v-kit Hardy-Zuckerman 4 feline sarcoma viral oncogene homolog)	5′-GGCATCAGGGTGACTTCAAT-3′	59.93	128	NM_001044525.1
	5′-GGTGGTTGTGACATTTGCAG-3′	60.01		
NANOG (nanog homeobox)	5’-ATCCAGCTTGTCCCCAAAG-3’	57.32	438	NM_001129971.1
	5’-ATTTCATTCGCTGGTTCTGG-3’	56.40		
OCT4 (POU class 5 homeobox 1)	5’-AGGTGTTCAGCCAAACGACC-3’	60.82	335	NM_001113060.1
	5’-TGATCGTTTGCCCTTCTGGC-3’	60.96		
**Early cardiac differentiation-related genes**				
MEF2C (myocyte enhancer factor 2C)	5′-TGATCAGCAGGCAAAGATTG-3′	59.95	112	NM_001044540.1
	5′-AGTGAGCTGACAGGGTTGCT-3′	60.06		
GATA-4 (GATA binding protein 4)	5’-TCTCGGAAGGCAGAGAGTG-3’	58.43	191	NM_214293.1
	5’-GCAGTTGGCACAGGAGAGG-3’	60.67		
**Hematopoietic-related gene**				
CD34 (CD34 molecule)	5’-GGAAACCACACCAGATGCTT-3’	58.38	164	NM_214086.1
	5’-AGGTCTGAGGCTGGACAGAA-3’	60.18		
**Mature cardiomyocytes-related genes**				
CX43 (gap junction protein, alpha 1, 43kDa)	5’-CACCAGGTGGACTGTTTCCT-3’	59.53	151	NM_001244212.1
	5’-TCTTTCCCTTCACACGATCC-3’	57.24		
TNNI3 (troponin I type 3 (cardiac))	5′-ATGCCCGCGTGGACAAGGTG-3′	59.97	133	NM_001098599.1
	5′-CGCAGGGTGGGCCGCTTAAA-3′	59.97		
ACTC1 (actin, alpha, cardiac muscle 1)	5′-CTTCCAACCCACCCTTCTTT-3′	60.33	120	NM_001170517.2
	5′-GTTGCAAGTCCTGGTCTGGT-3′	60.16		
**Growth factors-related genes**				
VEGFA (vascular endothelial growth factor A)	5′-ATCTTCAAGCCGTCCTGTGT-3′	59.73	145	NM_214084.1
	5′-TCTCTCCTATGTGCTGGCCT-3′	59.97		
IGF-1 (insulin-like growth factor 1)	5′-GACGCTCTTCAGTTCGTGTG-3′	59.62	141	NM_214256.1
	5′-CTCCAGCCTCCTCAGATCAC-3′	59.94		
IGF-1R (insulin-like growth factor 1 receptor)	5′-CAGTCCTAGCACCTCCAAGC-3′	60.01	134	NM_214172.1
	5′-GTCTTCGGCCACCATACAGT-3′	60.00		
HGFL (hepatocyte growth factor-like protein homolog)	5′-GGGGACGATACTGTCCTGAA-3′	59.93	109	XM_001924610.1
	5′-GTCCCTCAGTGCACATCTCA-3′	59.83		
FGFR2 (fibroblast growth factor receptor 2)	5′-AAACACGTGGAAAAGAACGG-3′	60.01	118	NM_001099924.1
	5′-TCACATTGAACAGAGCCAGC-3′	59.99		
TGFB1 (transforming growth factor, beta 1)	5′-TTAACGGGTTCAATTCTGGC-3′	59.94	145	NM_214015.1
	5′-TAGTTGGTATCCAGGGCTCG-3′	60.09		
**Housekeeping gene**				
ACTB (actin, beta)	5′-TGCGCAGAAAATGAGATGAG-3′	60.10	136	AY550069.1
	5′-CACCTTCACCGTTCCAGTTT-3′	60.01		

Conventional PCR amplification was performed using the Taq DNA Polymerase Recombinant kit (Invitrogen) in a PXE 0.2 thermocycler (Thermo). Gene expression levels were analyzed and normalized with the Gene Tools software (Synoptics Limited) using beta-actin (ACTB) as a housekeeping gene. The relative quantification was made by measuring the brightness intensity of each band using the GeneSnap software.

### Adipogenic, chondrogenic and osteogenic differentiation of porcine cardiosphere-derived cells

The differentiation of CDCs was performed when the cells reached 80% of confluence with media replacement every third day. Standard published protocols were used to promote osteogenic [21], adipogenic [22] and chondrogenic differentiation [23]. Oil Red O Staining was performed for adipogenic cultures, the Alcian Blue 8GX staining was performed for chondrogenic cultures and Alizarin Red S staining was performed for osteogenic differentiation. Differentiated cells were observed by optical microscopy.

### Myocardial infarction model creation

Four Large White pigs were housed in the animal facility at Minimally Invasive Surgery Centre and used for all experimental procedures. Animals were aged between 3–4 months and weighed between 30–35 kilograms. Animal care and all experimental procedures were approved by the ethics committee for animal research of the local government. All surgical procedures were performed under anesthesia, and all efforts were made to minimize suffering. Each animal was premedicated with diazepam 0.1 mg/kg, ketamine 10 mg/kg, and atropine 0.01 mg/kg intramuscularly. Intravenous hydration with normal saline was established by catheterization of the auricular vein with 18–22 gauge needles (Abbott) and maintained during procedures. Induction of anesthesia was performed intravenously with 2 mg/kg of propofol. After the pig was endotracheally intubated, it was connected to a system for anesthesia (Ohmeda Excel 210) and a mechanical ventilator Ohmeda 7800 (Ohmeda). Anesthesia was maintained with 2.0%%–2.5% halothane, and blood pressure, electrocardiogram, O_2_ saturation, and end tidal CO_2_ were monitored closely throughout the procedure. The pigs were fixed on the operating table in the supine position with cranial and caudal extension of the limbs. The thorax and upper abdomen were shaved and draped in a sterile fashion. Continuous infusion of lidocaine at rate of 1 mg/kg/h (Braun Medical) was used through the procedure. Systemic heparin was injected intravenously (150 UI/kg) prior to percutaneous sheath placement. Under aseptic conditions, a right femoral arterial access was established using the Seldinger technique and a 7 Fr introducer sheat (Terumo) was placed percutaneously into the femoral artery. Under fluoroscopic guidance (Philips Mobile Digital Angiographic System-BV Pulsera, Philips Medical System), a 6 Fr hockey stick guiding Mach 1 catheter (Boston Scientific Corporation) was introduced and placed at the origin of the left coronary artery. Coronary angiograms were obtained in the 40° left anterior oblique projection to better demonstrate the length of the Left Anterior Descending artery (LAD), and a 0.0014 coronary Hi-torque guidewire (Abbott Vascular) was advanced inside the LAD. After measuring the diameter of the LAD immediately below the origin of the first diagonal, an over-the-wire PTCA balloon of appropriate diameter (typically 3mm) (Apex OTW, Boston Scientific) was advanced to this location and inflated to occlude the LAD flow for 90 min. A lidocaine bolus was also administered immediately before balloon inflation and deflation. Upon balloon deflation, the coronary artery was checked for patency by repeating angiogram. Animals were maintained fully monitored under general anesthesia for 45 min after infarct induction, in order to treat any malignant arrhythmias that may ensue.

### Magnetic Resonance Imaging (MRI)

**Fig 1:**
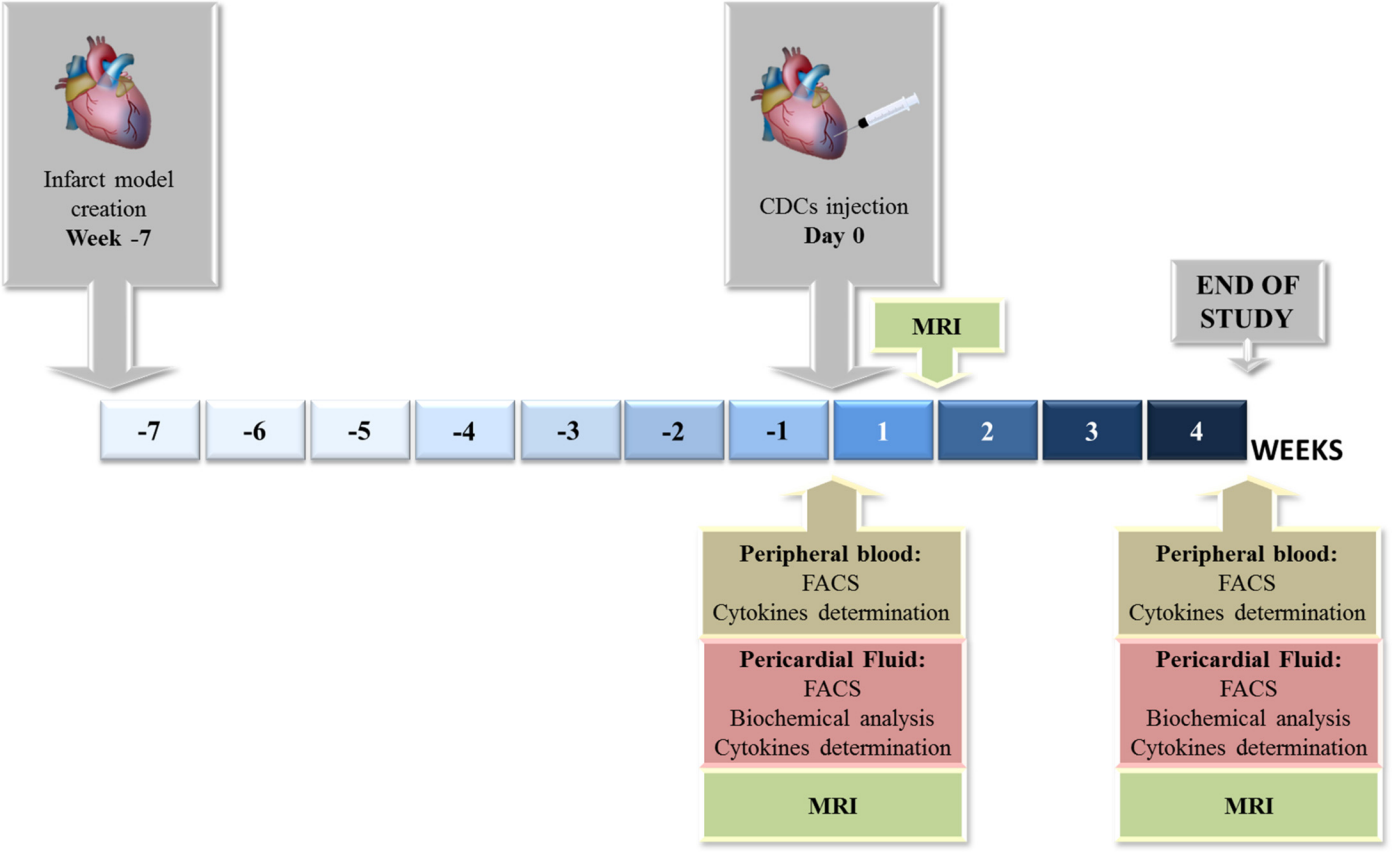
Experimental design. Seven weeks after infarct model creation, CDCs were intrapericardially injected. 30 days after CDCs administration, animals were euthanized. MRI was performed on days 0 (before CDCs administration), 7 and 30. On days 0 and 30, blood and pericardial fluid samples were collected for flow cytometry, biochemical analysis and cytokine determinations.

Cardiac MRI was performed before the creation of the porcine infarct model, 7 weeks post-myocardial infarction (just before CDCs administration), and then at day 7 and 30 post-CDCs administration (see Fig 1), using a 1.5 T MR system (Intera 1.5T Philips Medical System). All imaging was performed under general anesthesia using retrospective cardiac gating with the animal in sterna decubitus and a four elements phase array coil was placed around the animal chest. Images were acquired in the intrinsic cardiac planes: short axis, long axis and four chamber views. For measurement of ventricular function and mass breath hold balanced SSFP, cine images were obtained over the entire ventricle. For infarct size measurements, images were acquired 5–15 min after the injection of 0.2 mmol/kg of a gadolinium-based contrast agent using a breath hold 3D gradient-echo inversion-recovery sequence. MR images were analyzed for ejection fraction, end diastolic and systolic volumes, mass and infarct size. In order to perform a robust comparison and avoid the influence of the animal’s growth on the results, volume data were indexed y Body Surface Area (BSA), using the weight-based formula described by Kelley [24]. All determinations were performed by an investigator blinded to the group allocation.

### Intrapericardial administration of CDCs

Seven weeks after myocardial infarction model creation, each animal was pre-medicated with diazepam 0.3 mg/kg and ketamine 10 mg/kg intramuscularly. Intravenous hydration with normal saline was established by catheterization of the auricular vein with 18–20 gauge needles (Abbott) and maintained during procedures. Induction of anesthesia was performed intravenously with 2 mg/kg of propofol. After the pig was endotracheally intubated, it was connected to a system for anesthesia (Leon Plus, Heinen+Löwenstein). Anesthesia was maintained with 1.8%–2% sevofluorane, and blood pressure, electrocardiogram, O_2_ saturation, and end tidal CO_2_ were monitored closely throughout the procedure. The pigs were fixed on the operating table and thorax and upper abdomen were shaved and draped in a sterile fashion. An injection of 300,000 CDCs/kg in 5 mL of Hypothermosol (BioLife Solutions) was performed via thoracotomy using an Abbocath®-T 20G catheter (Hospira). The incision was closed in layers and the animals were allowed to recover.

### Tissue sampling

The animals were euthanized 4 weeks post-CDCs administration by a lethal dose of potassium chloride (1–2 mmol/kg) while under deep anesthesia, as recommended by the American Veterinary Medical Association (AVMA Guidelines for the Euthanasia of Animals: 2013 Edition. Available at: https://www.avma.org/kb/policies/documents/ euthanasia.pdf). The hearts were firstly examined *in situ*. Gross visual inspection was focused on possible complications associated with the procedures and potential damages to the pericardium, epicardium and surrounding structures in the mediastinum.

### Biochemical analysis of pericardial fluid and plasma

Before intrapericardial administration of CDCs, blood and pericardial fluid (PF) samples from animals were collected. The PF was aspirated from the pericardial cavity using an Abbocath®-T 20G catheter. The PF was centrifuged for 5 min at 450 x g. To determine their biochemical composition (albumin, alkaline phosphatase, total bilirubin, cholesterol, creatinine, gamma-glutamyl transferase, glucose, glutamic oxaloacetic transaminase, glutamic-pyruvic transaminase, HDL cholesterol, LDL cholesterol, reactive C protein, total proteins, triglycerides, urea and calcium concentrations), the pellet was used for FACS analysis and the supernatants for subsequent biochemical analyses were centrifuged and passed through a 0.22 μm filter to remove cell debris. Plasma samples and PF supernatants were processed in the random access clinical analyzer Metrolab 330 (Metrolab S.A.). At day 30 after CDCs administration, these sampling and determinations were repeated and the initial and final measurements were compared.

### Cytokines analysis

The pericardial fluid supernatants and plasma samples were stored at -80°C until further processing. Cytokines levels of IFNα, IFNγ, IL-1b, IL-10, IL-12p40, IL-4, IL-6, IL-8 and TNFα were analyzed using the Luminex xMAP technology, a multiplexed sandwich immunoassay. The measurements were determined using the ProcartaPlex Porcine Cytokine & Chemokine Panel 1 (catalog number EPX090-60829-901) according to the manufacturer’s instructions (eBioscience). The concentrations of the different cytokines were expressed as pg/mL, and calculated according to a standard curve.

### Flow cytometry analysis of peripheral blood lymphocytes and pericardial fluid cells

Peripheral blood lymphocytes (PBLs) were isolated from blood samples collected before intrapericardial administration of CDCs (7 weeks post-infarction) and before euthanasia (30 days after intrapericardial CDCs administration). PBLs were obtained by centrifugation over Histopaque-1077 (Sigma) and washed twice with PBS. The PBLs were frozen and stored in liquid nitrogen. For *in vitro* experiments, cell aliquots were thawed at 37°C, added to 10 mL of DMEM and centrifuged at 1500 rpm for 5 min to eliminate DMSO. Pellet was resuspended in PBS for immediate FACS analysis.

For flow cytometric analysis of PBLs and pericardial fluid cells, the cells were resuspended in PBS containing 2% FBS and stained with monoclonal antibodies against porcine CD3, CD4, CD8 and CD16 (Serotec). The cytometric analysis was performed as follows: 2×10^5^ cells were incubated for 30 min at 4°C with appropriate concentrations of monoclonal antibodies. The cells were washed and resuspended in PBS. The flow cytometric analysis was performed on a FACScalibur cytometer (BD Biosciences) after acquisition of 10^5^ events. Cells were primarily selected using forward and side scatter characteristics. The percentage of CD4^+^ T cells (CD3^+^ CD4^+^), CD 8^+^ T cells (CD3^+^ CD8^+^), NK cells (CD3^-^ CD16^+^) and CD8^+^ T cells expressing CD16 (CD16^+^ on gated CD3^+^ CD8^+^ cells) were analyzed using CellQuest software (BD Biosciences). Isotype-matched negative control antibodies were used in all the experiments.

### Statistical analysis

Data were statistically analyzed using the Mann Whitney U test for variables with no parametric distribution. All *p*-values ≤ 0.05 were considered statistically significant. All the statistical determinations were made using SPSS-21 software (SPSS, Chicago, IL, USA).

## Results and Discussion

The appropriate route for cell administration is a fundamental step for the success of cardiovascular stem cell-based therapies. Many clinical trials are being conducted using different administration routes and several advantages or disadvantages have been attributed to any of these routes. In the clinical setting of myocardial infarction, percutaneous coronary intervention is routinely performed since early reperfusion therapy for occluded coronary arteries is the main therapeutic strategy. So, intracoronary stem cell therapy is easily and widely applied, compared to other delivery methods [25]. Actually, endoluminal strategies are especially suited for the treatment of myocardial infarct in the acute phase, which is coincident with a high expression of chemoattractants and cell adhesion molecules [26]. However, intracoronary delivery has certain disadvantages: the immediate retention of cells is low, presumably because of rapid wash out of cells. Moreover, microvascular occlusion can occur when large cells such as MSCs, or CDCs are infused [27,28]. Another option here would be the intravenous infusion, which is the most simple method for stem cell delivery, but its retention rate is very low [29].

On the other hand, intramyocardial injection has been considered to be an optimal route for cell delivery in patients with chronic myocardial ischemia which corresponds to a low expression of cell homing signals such as chemoattractants and cell adhesion molecules. This administration route appears to have a higher retention rate but there is a significant loss of transplanted cells due to myocardial contraction [30]. Moreover, intramyocardial administration into ischemic or scarred myocardium could create clusters of cells isolated from the normal architecture and therefore with limited blood supply, which could lead to poor cell survival [26]. For these reasons, different delivery routes could provide the answer to different clinical scenarios.

Intrapericardial administration could help to overcome the disadvantages of intramyocardial injection, since there is no aggression to the myocardium and the distribution of cells is homogeneous. Moreover, similarly to intramyocardial injection, this administration route would only be feasible on stable patients, and therefore not recommended in the acute setting, since it involves a surgical procedure which necessarily requires cardiovascular stability. In summary, an acute myocardial infarction model where the cells are administered at an early stage would not be the more suited for intrapericardial administration; on the contrary a more chronic scenario may provide the ideal setting in terms of stability and safety.

Our research group has recently evaluated the biodistribution pattern of intrapericardially administered MSCs in a clinically relevant large animal model. Our studies were performed on infarcted and non-infarcted animals and *in vivo* tracking of intrapericardially administered MSCs was performed by magnetic resonance imaging (MRI). In this study, we demonstrated that intrapericardial administration is a safe route for MSCs transplantation. This route has the great advantage of transferring relatively large amounts of cells without adverse effects. Moreover, the pericardial fluid provided an optimal environment for maintaining cell viability [10].

Once demonstrated the safety and biodistribution of intrapericardially administered cells, one important question to be answered was whether or not the intrapericardial administration of stem cells could mediate a local immunomodulatory effect. In this sense, the main objective of this work has been mainly focused on the evaluation of different immunological parameters in a clinically relevant animal model of acute myocardial infarction.

**Fig 2:**
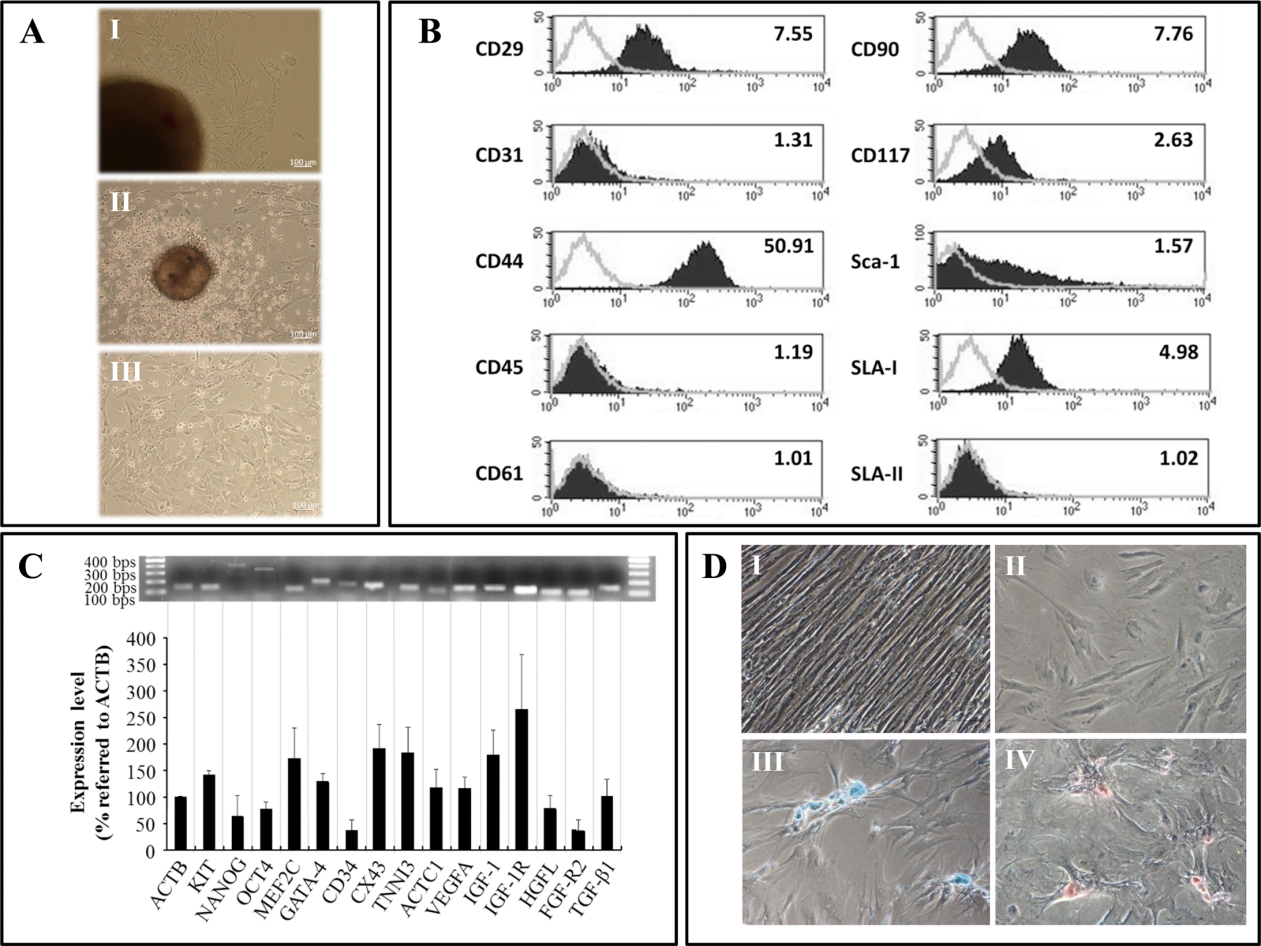
Characterization of cardiosphere-derived cells. CDCs were isolated from cardiac tissue explants of healthy pigs. The figure A shows explants in culture with some fibroblast-like cells migrating from them (A.I), cardiospheres with CDCs migrating from them (A.II) and CDCs in culture (A.III). Figure B shows the phenotypic analysis of CDCs by flow cytometry. Representative histograms together with the expression levels are shown. The expression level of cell surface markers is represented as Mean Relative Fluorescence Intensity (MRFI), which is calculated by dividing the Mean Fluorescent Intensity (MFI) (black lined histogram) by its negative control (grey lined histogram). Figure C corresponds to gene expression analysis by conventional RT-PCR. Mean ± SD of three different experiments are shown. Data are expressed as expression percentage referred to ACTB, used as control. The relative quantification was made by measuring the brightness intensity of each band with GeneSnap software. A representative image of one of the experiments is shown above. Figure D shows the differentiation potential of CDCs. Cells were maintained for 21 days with standard medium (control) (D.I) or with specific differentiation media for adipogenic, chondrogenic and osteogenic lineages. Differentiation was evidenced by specific stainings: Oil Red O for adipocytes (D.II), Alcian Blue for chondrocytes (D.III) and Alizarin Red S for osteocytes (D.IV).

Our first sets of experiments were conducted in the isolation, expansion and characterization of porcine cardiosphere-derived stem cells (CDCs). These cells were obtained from cardiac tissue explants and the stromal-like cells migrating from the explants (Fig 2A) were expanded according to standardized protocols described by the group of Marban et al. [12]. In contrast to our previous study where bone marrow derived stem cells were intrapericardially administered for the evaluation of safety and biodistribution of stem cells, here we have chosen CDCs because of their highly promising results reported from the first clinical trials. These clinical trials are currently being conducted using autologous CDCs in the setting of heart failure, either after coronary artery bypass grafting [31,32] (SCIPIO trial, NCT00474461) or after coronary stenting [17,18] (CADUCEUS trial, NCT00893360).

The phenotypical characterization of porcine CDCs by flow cytometry (Fig 2B), RT-PCR analysis (Fig 2C) and differentiation assays (Fig 2D) demonstrated that these cells were similar or at least equivalent to those porcine CDCs described by the group of Marban et al [12]. In the phenotypic characterization by flow cytometry they resulted positive for the expression of CD29, CD44, CD90, CD117, Sca-1 and SLA-I, and negative for the expression of CD31, CD45, CD61 and SLA-II. In the PCR analysis, they showed a positive expression of multipotential markers, early cardiac differentiation markers and mature cardiomyocytes markers, as well as different growth factors and their receptors. Finally, differentiation assay demonstrated their differentiation behaviour towards adipogenic, chondrogenic and osteogenic lineages.

Our porcine CDCs were *in vitro* expanded and detached from culture flasks at the day of intrapericardial administration. This allowed us to ensure the viability and the maintenance of proliferative behaviour of intrapericardially administered cells. To ensure the safety of our approach, we previously performed a pilot study in infarcted swine. The cardiac MRI did not show any evidence of adverse effects or cardiac toxicity when cells were intrapericardially administered at day 7 post-infarction (data not shown).

Non-adverse effects were noted during the surgical procedure and euthanized animals showed a normal conformation of tissues, with few pericardial adherences to thoracic wall due to the surgical intervention (data not shown). Considering that safety is a major issue in cardiovascular stem cell therapy, our findings suggest that intrapericardial delivery is a safe route for transplantation of allogenic CDCs. These results are in agreement with previous reports where the combined usage of adult stem cells and different biomaterials were safely administered by this route [33,34].

**Fig 3:**
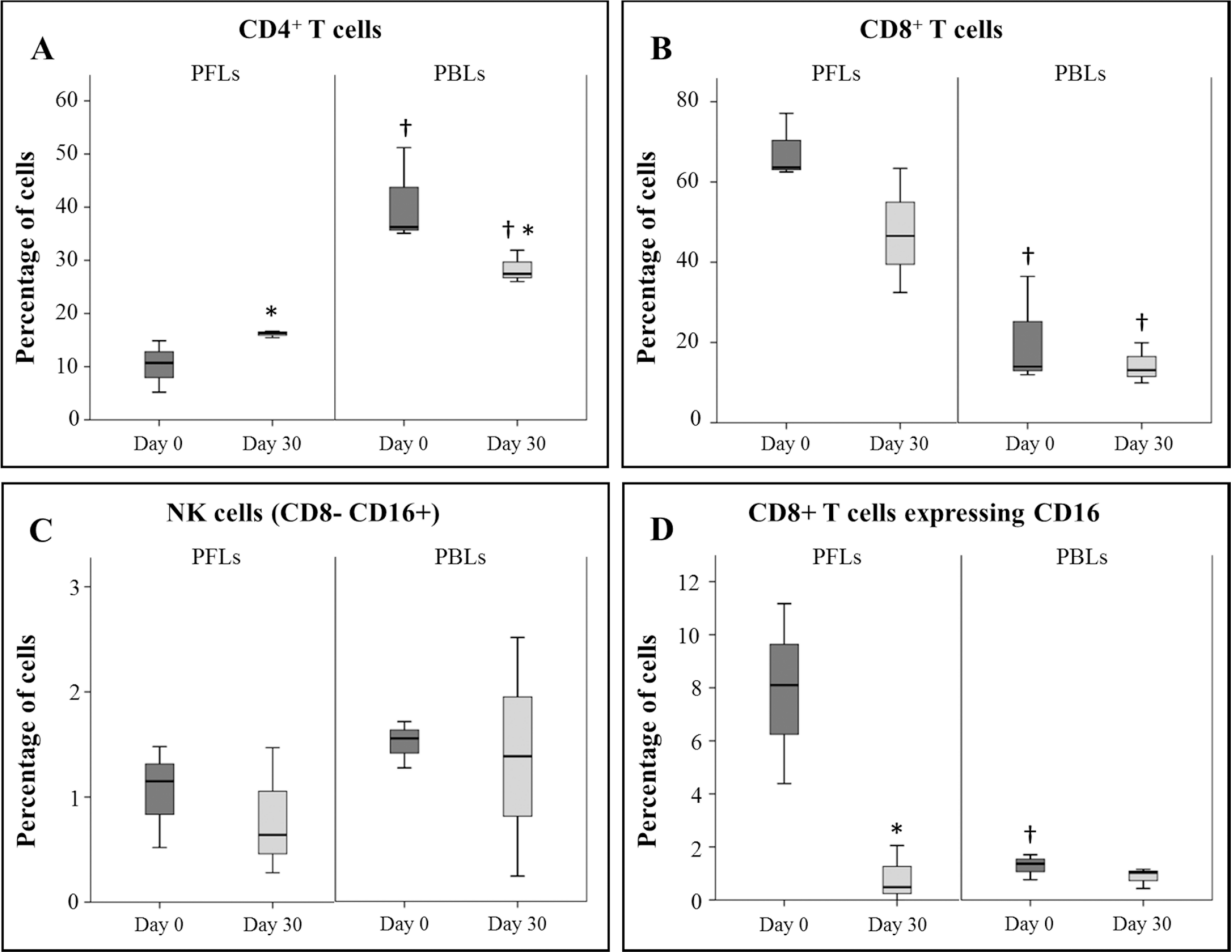
Lymphocyte subsets distribution in peripheral blood and pericardial fluid. Pericardial fluid lymphocytes (PFLs) and peripheral blood lymphocytes (PBLs) were collected before CDCs administration and 30 days post-administration for flow cytometry analysis. * Statistically significant differences (*p*<0.05) between different time points (n = 4). † Statistically significant differences (*p*<0.05) between PFLs and PBLs in the same time point (n = 4).

Once confirmed the safety aspects of intrapericardial administration using porcine CDCs, we aimed to compare the phenotype profile of resident lymphocytes in the pericardial fluid immediately before CDCs administration and 30 days post-administration (Fig 3). In this analysis, our interest was mainly focused in the quantification of lymphocyte subpopulations and the expression of lymphocyte activation markers. The statistical comparison of pericardial fluid lymphocytes showed that the percentage of CD4^+^ T cells was significantly increased after CDCs administration (Fig 3A). However, CD8^+^ T cells and NK cells were unaffected by the intrapericardial administration of CDCs (Fig 3B and 3C). Previous reports have demonstrated an induction of CD4^+^CD25^+^Foxp3^+^ Tregs by allogeneic administration of MSCs [35]. Our current results may indicate that the expanded subset of CD4 T cells may correspond to *in vivo* expanded Tregs, however, this hypothesis should be further confirmed by RT-PCR.

The analysis of activation markers in lymphocyte subsets was focused on the expression of CD16 protein in the surface of CD8^+^ T cells. Here we showed that, the percentage of CD8^+^ T cells expressing CD16 in the pericardial fluid was significantly decreased after intrapericardial CDCs administration (Fig 3D). These CD8^+^ T cells expressing CD16 have been considered as terminally differentiated effector T cells [36] and positive for perforin as specifically described in pigs [37]. Based on these results, here we hypothesize that the increased percentage of CD8^+^ T cells expressing CD16 in the pericardial fluid (measured at 7 weeks post-infarction) could be the consequence of inflammatory microenvironment linked to myocardial infarction. Moreover, the significant decrease of CD8^+^ T cells expressing CD16 towards similar levels (measured at day 30 post-administration) would be the consequence of the immunomodulatory activity of CDCs exerted under inflammatory conditions [38].

In bibliography, it has been demonstrated in a large cohort of patients that activated lymphocytes with a “special immunophenotype” are frequently found in the pericardial fluid of patients undergoing open cardiac operation with different forms of heart disease [39]. Our results are in agreement with this observation. Indeed, here we found that the percentage and phenotype of CD4^+^ and CD8^+^ T cells in the pericardial fluid of infarcted animals is significantly different to the percentage and phenotype of CD4^+^ and CD8^+^ T cells in peripheral blood (Fig 3A and 3B).

Additional biochemical parameters were studied in pericardial fluids from infarcted animals prior to CDCs administration and 30 days post-administration. Most of the biochemical parameters were unaffected by the intrapericardial administration of CDCs and significant differences were only observed in the quantification of alkaline phosphatase, creatinine, gamma-glutamyl transferase (GGT) and total proteins (Table 2). The alkaline phosphatase is present in many human tissues and has been defined as a well-known marker for bone marrow-derived MSCs and embryonic stem cells [40–42]. Possibly, the increase of alkaline phosphatase in the pericardial fluid could be interpreted as a consequence of the presence of CDCs in the pericardial fluid. Apart from alkaline phosphatase, an augmentation of total protein was also found in pericardial fluid after CDCs administration. This increase is probably due to the presence of transferred cells, or to the paracrine-released proteins from these cells. Finally, it is important to discuss the decrease of GGT observed in the pericardial fluid. The levels of GGT in serum have been used to predict coronary heart disease in a large cohort of patients, especially, a stronger association was found in subjects aged less than 60 years [43]. The GGT levels have been shown to be a predictive marker in the development of cardiovascular disease [44]. Moreover, there is a relationship between serum GGT level and coronary blood flow [45] and a clear association between this enzyme with severity of heart failure [46]. According to these clinical findings, and taking into account that here we quantified CGT in the pericardial fluid (not in serum), we could assume that this significant decrease could be considered as a good prognosis indicator that may reflect an improvement of heart function.

**Table 2:** Biochemical analysis of pericardial fluid before and after CDCs administration.

	Pericardial fluid	
	Pre-CDCs	Post-CDCs
**Albumin (g/dL)**	0.84± 0.23	1.09± 0.42
Alkaline phosphatase (U/L)*	14.00± 5.29	20.67± 6.03
**Total bilirubin (mg/dL)**	0.03± 0.03	0.06± 0.01
**Cholesterol (mg/dL)**	9.67± 4.62	12.33± 6.81
Creatinine (mg/dL)*	1.35± 0.26	1.83± 0.35
GGT (U/L)*	24.33± 10.02	15.33± 6.35
**Glucose (mg/dL)**	91.67± 5.77	86.33± 1.53
**GOT (U/L)**	12.33± 2.08	13.33± 8.50
**GPT (U/L)**	0.00± 0.00	0.50± 0.71
**HDL cholesterol (mg/dL)**	5.17± 1.67	5.73± 1.79
**LDL cholesterol (mg/dL)**	7.49± 1.81	8.60± 4.14
**CRP (mg/L)**	0.39± 0.54	0.70± 0.46
Total protein (g/dL)*	1.73± 0.45	2.18± 0.60
**Triglycerides (mg/dL)**	5.00± 2.00	11.67± 8.14
**Urea (mg/dL)**	17.40± 8.62	22.53± 3.34
**Calcium (mg/dL)**	4.60± 1.04	5.17± 1.71

GGT: gamma-glutamyl transferase; GOT: glutamic oxaloacetic transaminase; GPT: glutamic-pyruvic transaminase; CRP: C-reactive protein.

*p ≤0.05 in a paired Student's *t*-test (n = 4).

Apart from the analysis of leukocyte subsets and biochemical parameters in the pericardial fluid, here we aimed to determine the effect of intrapericardial administration of CDCs in the cytokine microenvironment. The limited availability of porcine-specific monoclonal antibodies did not provide a deep analysis of immune status in the pericardial fluid. Besides of this limitation, here we aimed to quantify a commercially available panel of TH1/TH2 cytokines in pericardial fluid and plasma samples from infarcted animals with intrapericardially administered CDCs. These samples were obtained from animals immediately before CDCs administration and 30 days post-intrapericardial administration. Again, the limited availability of porcine-specific reagents for cytokines was an important limitation and Luminex technology allowed us the quantification of only nine cytokines. The following panel of cytokines was measured: IL-1beta, IL-4, IL-6, IL-8, IL-10, IL-12p40, IFN-alpha, IFN-gamma and TNF-alpha.

**Fig 4:**
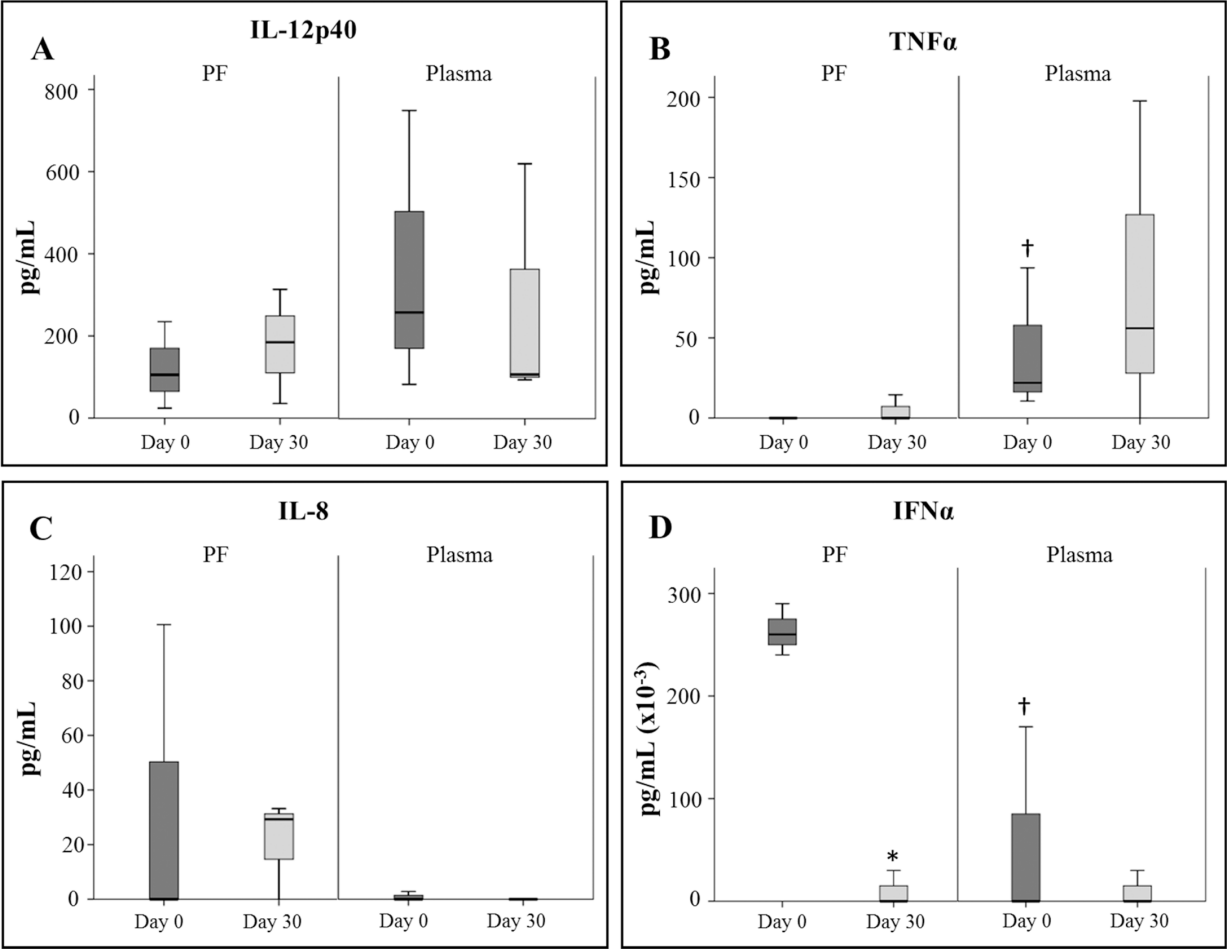
Cytokines levels in pericardial fluid and plasma samples. Cytokines levels were determined before CDCs administration and 30 days post-administration using the Luminex xMAP technology. * Statistically significant differences (*p*<0.05) between different time points (n = 4). † Statistically significant differences (*p*<0.05) between PF and plasma in the same time point (n = 4).

Our results demonstrated that, only four out of nine cytokines were detectable by this method. In comparison to plasma samples, the IL-12p40 was found to be less abundant in pericardial fluid. The IL-12p40 levels only showed a trend to increase (*p* = 0.06) when pericardial fluids were compared (pre- and post- CDCs administration) (Fig 4A). The IL-12p40 is a component of IL-12 and IL-23, so the increase of this protein in the pericardial fluid may provide a negative feedback by competitively binding to the IL-12 receptor [47].

Similarly to IL-12p40, the TNF-alpha was more abundant in plasma than in pericardial fluids and no differences were found between pre- and post-administration. There were non-statistical differences when compared plasma and pericardial fluid samples (Fig 4B).

Contrarily to IL-12p40 and TNF-alpha, the IL-8 was found to be more abundant in pericardial fluid than in plasma (Fig 4C). These results are in agreement with similar studies performed in patients undergoing coronary artery surgery [48]. Moreover, considering that this chemokine has been linked to a local inflammatory process in patients with pericardial effusions [49], the presence of this chemokine could be the consequence of inflammatory response usually linked to an acute myocardial infarct [50].

Finally, our results showed a very significant difference when IFN-alpha levels were compared in pericardial fluids. Indeed, the IFN-alpha level was significantly reduced in after intrapericardial administration (Fig 4D). This cytokine is known to be secreted by fibroblasts, monocytes, macrophages, dendritic cells, natural killer but also by T cells [51] and the significant decrease of this cytokine in the pericardial sac could be the reflection of an overall improvement in terms of inflammatory activation. The association between IFN-alpha and pericarditis has been reported in clinical settings where the pericarditis was related to interferon alpha therapies [52–54].

**Fig 5:**
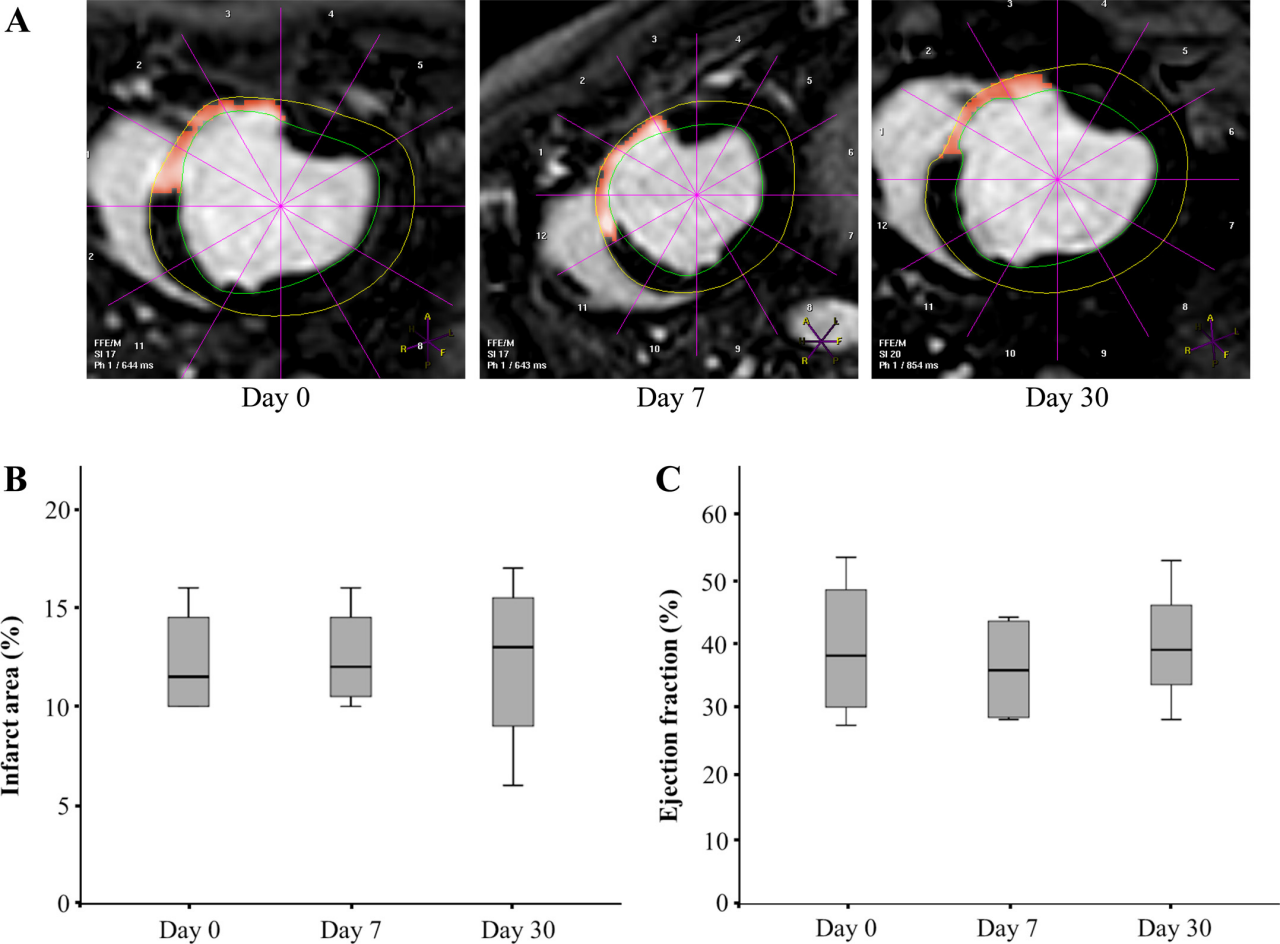
Cardiac magnetic resonance imaging. Cardiac function was measured with cardiac magnetic resonance imaging. The panel A represent a representative image of the measurement of the thickness of the infarcted (septum) and healthy lateral free wall in end diastolic short axis views. B and C graphics represent the infarct area and the ejection fraction measurements, respectively, obtained on days 0 (before CDCs administration), 7 and 30. The lower boundary of the box indicates the 25th percentile and the upper boundary the 75th percentile. Bars above and below the box indicate the 90th and 10th percentiles. The line within the box marks the median. No statistically significant differences were found between groups (n = 4).

Regarding to the evolution of cardiac function parameters, magnetic resonance imaging was performed at day 0 (7 weeks post-myocardial infarction), days 7 and 30 post-intrapericardial administration. This technique has been recently used by Malliaras et al. in porcine infarct models to evaluate stem cell therapies which confirmed the usefulness of this technique for monitoring regenerative efficacy [55]. Our results showed no significant differences among untreated animals (day 0) and treated animals in terms of cardiac parameters (Table 3 and Fig 5). Contrarily to the significant changes reported in the inflammatory status after intrapericardial administration of CDCs (both in phenotype of peripheral fluid lymphocytes and inflammatory cytokines), the absence of an improvement of cardiac function could be the consequence of using a 7 weeks infarcted animal. This model is considered as a chronic myocardial infarct model and the size and chronicity of the infarct becomes a serious obstacle to identify changes in the cardiac function. Future studies will be performed in order to adequate this therapy to the chronic infarct model, which provides the ideal settings for the study of this administration route in terms of stability and safety.

**Table 3:** Cardiac parameters calculated from MRI exams performed through the study.

	Day 0 (pre-administration)	Day 7	Day 30
**Weight (kg)**	43.50± 2.65	44.75± 1.89	57.25± 3.09
**EF (%)**	39.40± 11.40	36.20± 8.56	40.2± 12.23
**EDVi (mL/m**^**2**^**)**	120.63± 30.69	122.18± 34.72	106.30± 21.63
**ESVi (mL/m**^**2**^**)**	75.70± 32.01	80.03± 32.24	65.18± 26.04
**% Infarct**	12.25± 2.87	12.5± 2.64	12.25± 4.65
**Infarct mass (g)**	10.18± 4.05	10.92± 4.38	8.73± 4.12

Data presented as mean ± standard deviation (n = 4). EF: Ejection fraction. EDVi: End diastolic volume indexed to body surface area. ESVi: End systolic volume indexed to body surface area.
